# Computer Viewing Model for Classification of Erythrocytes Infected with *Plasmodium* spp. Applied to Malaria Diagnosis Using Optical Microscope

**DOI:** 10.3390/medicina61050940

**Published:** 2025-05-21

**Authors:** Eduardo Rojas, Irene Cartas-Espinel, Priscila Álvarez, Matías Moris, Manuel Salazar, Rodrigo Boguen, Pablo Letelier, Lucia San Martín, Valeria San Martín, Camilo Morales, Neftalí Guzmán

**Affiliations:** 1 Laboratorio de Investigación en Salud de Precisión, Departamento de Procesos Diagnósticos y Evaluación, Facultad de Ciencias de la Salud, Universidad Católica de Temuco, Temuco 4780000, Chile; eduardo.rojas@uct.cl (E.R.); icartas@uct.cl (I.C.-E.); palvarez2020@alu.uct.cl (P.Á.); mmoris2020@alu.uct.cl (M.M.); manuel.salazar2020@alu.uct.cl (M.S.); rboguen@uct.cl (R.B.); pletelier@uct.cl (P.L.); lsanmartin@uct.cl (L.S.M.); valeria.sanmartin@uct.cl (V.S.M.); 2 Laboratorio SouthGenomics SpA, Temuco 4780000, Chile; 3 Centro de Investigación, Innovación y Creación UCT (CIIC-UCT), Universidad Católica de Temuco, Temuco 4780000, Chile; 4 Departamento de Procesos Terapéuticos, Facultad de Ciencias de la Salud, Universidad Católica de Temuco, Temuco 4780000, Chile; camilo.morales@uct.cl

**Keywords:** malaria, machine learning, convolutional neural network, artificial intelligence, blood morphology, diagnosis, precision medicine, laboratory medicine

## Abstract

*Background and Objectives:* Malaria is a disease that can result in a variety of complications. Diagnosis is carried out by an optical microscope and depends on operator experience. The use of artificial intelligence to identify morphological patterns in erythrocytes would improve our diagnostic capability. The object of this study was therefore to establish computer viewing models able to classify blood cells infected with *Plasmodium* spp. to support malaria diagnosis by optical microscope. *Materials and Methods:* A total of 27,558 images of human blood sample extensions were obtained from a public data bank for analysis; half were of parasite-infected red cells (*n* = 13,779), and the other half were of uninfected erythrocytes (*n* = 13,779). Six models (five machine learning algorithms and one pre-trained for a convolutional neural network) were assessed, and the performance of each was measured using metrics like accuracy (A), precision (P), recall, F1 score, and area under the curve (AUC). *Results:* The model with the best performance was VGG-19, with an AUC of 98%, accuracy of 93%, precision of 92%, recall of 94%, and F1 score of 93%. *Conclusions:* Based on the results, we propose a convolutional neural network model (VGG-19) for malaria diagnosis that can be applied in low-complexity laboratories thanks to its ease of implementation and high predictive performance.

## 1. Introduction

Malaria is a type of parasitosis caused by protozoa of the genus *Plasmodium* spp. present in tropical and subtropical regions [1]. According to the WHO, 249 million cases and 608,000 deaths were reported during 2022 in 85 countries where the disease is endemic, with Africa accounting for 94% of the confirmed cases [2]. Although the mortality rate of the disease has fallen, it is still considered one of the principal causes of death worldwide [3,4]. The complications described are important alterations in various organs as a consequence of the parasite invading the liver and the erythrocytes to complete its life cycle [5,6,7].

Although malaria can be diagnosed by rapid diagnostic tests or by detecting the parasite using molecular methods [3,8,9], these procedures must be carried out in specialized laboratories [10]. Optical microscopes are still considered to be the gold standard for diagnosis [11]. Although this is an accessible method for basic clinical laboratories, it depends on the observer’s expertise [8,11], which may have an impact on diagnostic capability.

In view of this difficulty, computerized microscope methods have recently become increasingly important [12,13]. The development of deep learning methodologies with convolutional neural networks (CNNs) has improved the results of parasite identification compared with traditional techniques [14]. These automatic learning tools, based on morphological characteristics and patterns, have proven themselves to be useful for diagnosing malaria [12,15,16].

The availability of open code data and images is crucial for establishing prediction models, since access to a large dataset is fundamental to ensure robust and reliable results [17,18]. Therefore, the object of this study was to compare the performance of different machine learning (ML) models (Support Vector Machine, Random Forest, Decision Tree, K-Nearest Neighbors and GaussianNB) and one pre-trained convolutional neural network (CNN) (VGG-19) in classifying erythrocytes infected with *Plasmodium* spp.

## 2. Materials and Methods

### 2.1. Construction of Dataset

**Figure 1:**
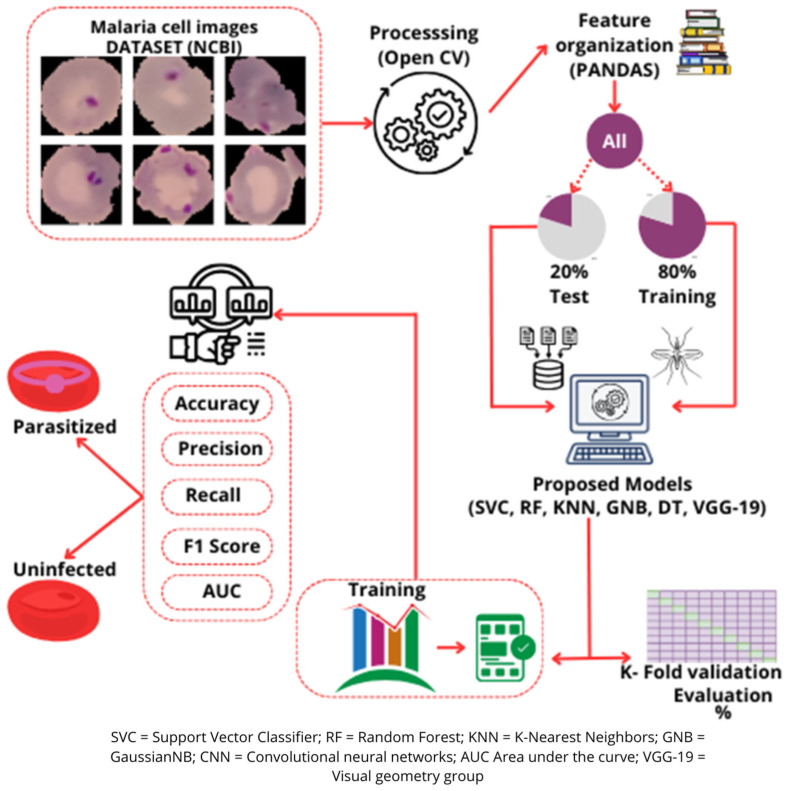
Flowchart for the processing and training of machine learning models.

The methodology used to train the machine learning models and the layered structure of the convolutional neural network (CNN) is described as follows. A public dataset containing May–Grunwald–Giemsa-stained blood smear images was downloaded from the Malaria Project of the National Library of Medicine (LHNCBC) (https://lhncbc.nlm.nih.gov/LHC-research/LHC-projects/image-processing/malaria-datasheet.html, accessed on 7 May 2024) for the retrospective analysis. A detailed overview of the workflow and CNN structure is provided in Figure 1. This dataset included 27,558 images of human blood extensions, of which half were red cells infected with the *Plasmodium* spp. parasite (*n* = 13,779) and the other half were uninfected erythrocytes (*n* = 13,779), as described by Kassim et al. [19]. The images were recorded using a high-resolution camera and reviewed and filed by an expert morphologist.

### 2.2. Training of Machine Learning (ML) Model

The images were processed using the Open Computer Vision (V 4.11.0) (OpenCV) and Numpy libraries (V 2.0.2), which enabled the morphological and textural characteristics of the blood cells to be extracted. OpenCV was used for pre-processing the images, keeping the pixel values at their original scale (0–255). Statistical characteristics were also calculated, such as the standard deviation, mean, and median of pixel intensity, together with the histogram of the image, divided into 8 bins to capture the distribution of the intensities. The textural characteristics were extracted by Discrete Wavelet Transformation (DWT).

All the data extracted from the information available in the images were organized in a structured dataset, with each row representing an image and each column a characteristic extracted. The final dataset included 23 characteristics per image and a class label (1 for parasite-infected erythrocytes and 0 for uninfected cells).

The dataset was divided by stratification to ensure that the classes (infected and uninfected erythrocytes) were balanced in the training and test sets. Eighty percent (*n* = 22,045) of the data were assigned to the training set and 20% (*n* = 5513) to the test set.

Five machine learning models were trained and evaluated: Support Vector Machine, Random Forest, Decision Tree, K-Nearest Neighbors (KNNs), and GaussianNB (GNB). The performance of each model was assessed in terms of its accuracy (A), precision (P), recall, F1 score, and area under the curve (AUC). K-5-Fold cross-validation was applied to all of the models. This method divides the training set into five subsets (folds) and trains/validates the model in each of them by iteration. Cross-validation was used to obtain a more robust estimate of the model’s performance, reducing the risk of over-demanding fit and ensuring that the results did not depend on a specific division of the data.

After cross-validation, each model was trained again using 100% of the training dataset. Finally, the performance of the models was assessed with the 20% test set, which had not been used in any previous stage, and a confusion matrix was created to analyze the performance of the various models in terms of false positives, false negatives, true positives, and true negatives.

### 2.3. Training a Convolutional Neural Network Model

Tensor Flow (2.18.0) and Keras (V 3.8.0) were used for image training and pre-processing. Initially, the images were normalized by scaling pixel values to the range 0 to 1, dividing each pixel value by 255, to create a more efficient model for computing purposes; the images were re-dimensioned to 224 × 224 pixels.

Data augmentation techniques were applied to improve the model’s capacity to generalize and manage variations in the images. These included the rotation of the images through different angles, horizontal and vertical displacement, and zoom to simulate different degrees of proximity. These transformations enabled the model to recognize infected and uninfected erythrocytes under varying conditions, reducing the risk of overfitting and improving robustness in real scenarios. This process was performed with the following parameters: rescale = 1./255, shear_range = 0.2, zoom_range = 0.2, and horizontal_flip = True. The dataset was stratified and divided into two parts (80% training and 20% test).

The network was based on the VGG-19 pre-trained model, described by Simonyan et al. [20], which includes convolution layers, Max Pooling and activation functions, and has produced adequate performance in medical images [21,22]. All convolutional layers in our implementation were frozen (layer.trainable = False) to preserve pre-trained weights and reduce overfitting. A custom classifier was added, consisting of a Flatten layer followed by a single Dense neuron with a sigmoid activation function, suitable for binary classification.

The model was optimized using the Adam algorithm [23] with a default learning rate of 0.001, and the loss function used was binary cross entropy. Recent studies have shown that using Adam can significantly improve precision and stability in classifying medical images [24,25]. Precision, accuracy, recall, F1 score, and AUC were used to assess the model. Furthermore, a confusion matrix was created to analyze the results in terms of false positives, false negatives, true positives, and true negatives.

The model was trained for 50 epochs using a batch size of 32. No explicit early stopping criterion was implemented. Local Interpretable Model–agnostic Explanations (V 0.2.0.1) (LIME) was used to check that the model focused on the correct area of the erythrocyte [26]. This method allows the model’s decisions to be interpreted by identifying the regions of the image which have the greatest influence on the classification. This interpretability approach strengthens the clinical trust in model decisions and ensures that predictions are based on meaningful morphological features rather than irrelevant artifacts or background noise.

### 2.4. Statistical Analysis

We compared the performance of the models in the assessment metrics used (accuracy, precision, recall, F1 score, and AUC) using the Friedman test followed by the Nemenyi post hoc test. The Friedman test is a non-parametric method for comparing multiple algorithms or models based on their performance in different sets of data or measurements [27]. It is useful when working with more than two models as it avoids the problems associated with multiple comparisons and does not assume a normal distribution of the data.

## 3. Results

A total of six models (five machine learning algorithms and one CNN) were assessed. K-5-Fold cross-validation was applied to all of the machine learning models, showing consistent, robust performance in all of the algorithms (results detailed in Table A1). The performance of the models was then assessed using the test set (20% of the dataset), the results of which are presented in Table 1. In the case of the VGG-19 model, 5-fold cross-validation was not performed in order to reduce the computational time associated with training deep neural networks.

The machine learning model which obtained the best performance was Random Forest, with A of 95.77%; P of 96.42%; R of 95.21%; F1 score of 95.81%; and AUC of 95.78%. The results observed for the VGG-19 convolutional neural network model were A of 93.14%; P of 92.04%; R of 94.22%; F1 score of 93.11%; and AUC of 97.76%.

**Figure 2:**
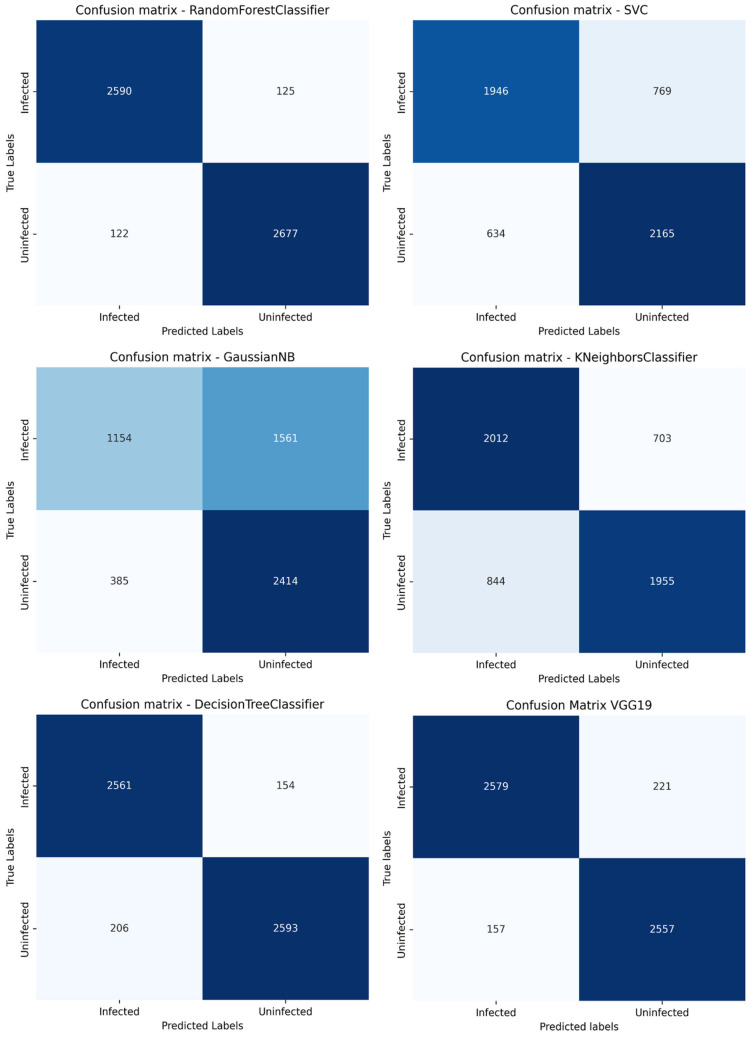
Confusion matrices of machine learning and CNN algorithms.

**Figure 3:**
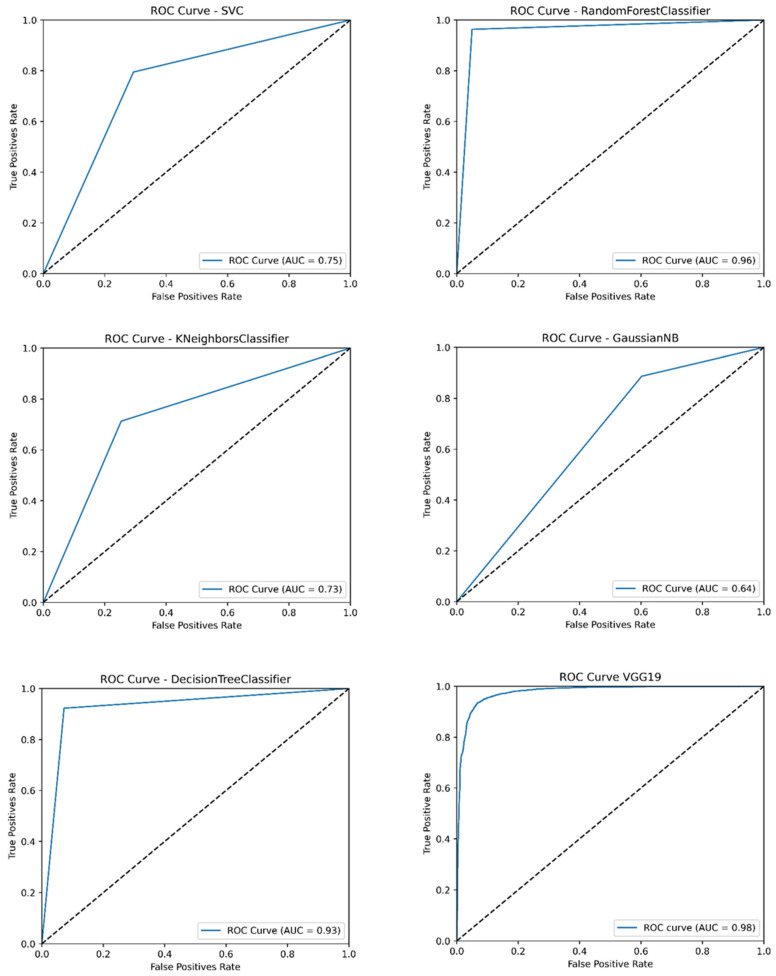
ROC curves of machine learning and CNN algorithms.

**Figure 4:**
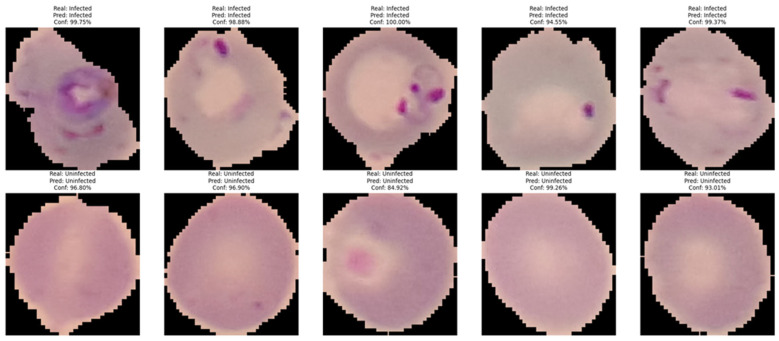
Classification and prediction by VGG-19 of infected and uninfected red cells.

**Figure 5:**
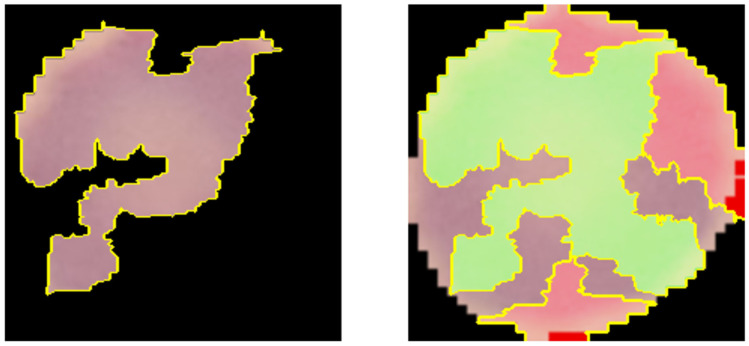
Result of the use of LIME to check the functioning of VGG-19. **Left**: segmentation of the erythrocyte on a black background highlighting the critical region identified by the model as influencing its decision most strongly. **Right**: the original erythrocyte. The areas colored red indicate the characteristics assigned most importance in the classification by the model. These regions contain morphological characteristics potentially associated with infection by parasites of the genus Plasmodium. In this case, the erythrocyte was classified as uninfected.

The confusion matrices for each model are shown in Figure 2. Random Forest presented the lowest results for false negatives and false positives. Figure 3 presents the ROC curves of each algorithm, showing the good performance of Random Forest (AUC 96%) and VGG-19 (AUC 98%). The real classification of infected and uninfected red cells, compared with the prediction by VGG-19, is presented in Figure 4, showing the performance of the model. Figure 5 presents the result of the application of LIME, indicating the area of the image evaluated to classify the red cell.

In general terms, both Random Forest and VGG-19 were able to correctly classify 95% of the cases, achieving a mean area under the curve (AUC) of 97%. In the Friedman test, no statistically significant differences were observed in the performance metrics between the models assessed.

## 4. Discussion

Optical microscopy is still considered the gold standard for diagnosing malaria. Although this is an accessible method for basic clinical laboratories, it depends on the observer’s expertise [8,11], which may have an impact on diagnostic capability, and the accurate, timely, and species-specific diagnosis of malaria is essential for successful treatment. Malaria diagnosis can be improved by the use of prediction models based on computer viewing methods. In this study, we assessed various computer viewing models for classifying erythrocytes infected with *Plasmodium* spp.

Among the machine learning models assessed, Decision Tree and Random Forest proved to be robust classification models, with better performance observed for Random Forest. These results are consistent with previous evidence, which has highlighted the effectiveness of these models in medical classification due to their ability to handle complex characteristics and heterogeneous data [28,29]. The precision achieved by the models indicates that they successfully select the right region of the erythrocyte to carry out the prediction.

Various studies have established neural network models for diagnosing malaria [14,15,16,17,30,31,32]; however, the present study is distinguished by employing an innovative approach through the use of the LIME method. This technique allowed us to verify the internal functioning of the trained model and provide interpretability and transparency to the decisions of the VGG-19 model. We consider this interpretability to be a valuable differential contribution to strengthen confidence in the clinical implementation of AI in malaria diagnosis, and we suggest that this type of analysis should be pursued in future research related to medical images.

Furthermore, the use of the Adam optimizer in our model significantly increased its computing efficiency. Adam combines the advantages of the AdaGrad and RMSProp algorithms, allowing for the rapid and efficient updating of the model’s parameters, even in large, varied datasets. This characteristic reduces the need for manual adjustments in the learning rate, making the model lighter and more adaptable for implementation in limited hardware systems, for example in low-complexity laboratories or those with limited resources. In comparison with techniques like stochastic gradient descent (SGD), Adam achieves faster, more stable convergence, making model training more cost-efficient.

The use of public data gave us access to a large number of images, enabling us to establish robust prediction models. A crucial aspect to consider for the application of our model is the staining method used in microscopic preparations. The dataset employed for training consisted exclusively of images of blood smears stained with May–Grunwald–Giemsa (member of the Romanowsky group of stains). Consequently, the VGG-19 model has learned to recognize morphological patterns and textural features as they appear under this specific stain. This highlights the importance of standardizing the staining method to May–Grunwald–Giemsa in clinical laboratories implementing this model to ensure its optimal performance. Despite this specificity, May–Grunwald–Giemsa staining is a standard and widely used technique in routine hematology and for malaria diagnosis in various settings, including low-complexity laboratories, due to its efficacy and relatively low cost. The study does, however, present some limitations, since the models were trained mostly on images of erythrocytes infected with annular forms of the parasite. The exclusion of advanced stages of *Plasmodium* spp. implies the need to increase the size of the dataset in future studies to include different phases of the parasite’s cycle.

This model can be used in low-complexity laboratories to identify the parasite in a blood smear under optical microscope, as it does not require operator expertise. Furthermore, the use of web platforms or mobile applications could help to maximize the practical implementation of this tool, as proposed by Breslauer et al. [33]. This approach would give laboratories with limited resources access to diagnosis support tools.

As part of future work, we envision evaluating other, more recent convolutional neural network architectures, such as those specifically optimized for mobile devices or systems with limited computational resources (e.g., MobileNet, EfficientNet). It would be essential to comparatively analyze the computational cost and inference efficiency of these models in low-complexity or resource-constrained settings, contrasting them with the performance and requirements of the proposed VGG-19 model. This would allow for identifying the most suitable architecture not only in terms of diagnostic accuracy but also in its practical feasibility for large-scale implementation in malaria-endemic regions.

## 5. Conclusions

The convolutional neural network model VGG-19 exhibits superior performance in terms of false positives and negatives, making it a potentially more reliable model in more complex clinical scenarios. Based on these results, we propose a convolutional neural network model for malaria diagnosis that can be applied in low-complexity laboratories thanks to its high predictive capacity. Additional training with images of more advanced infected stages will help to determine the different life cycle stages of the parasite and contribute to improving model efficiency.

## Figures and Tables

**Table 1:** Performance of machine learning and CNN models assessed for the diagnosis of erythrocytes infected with malaria parasites.

CNN	MACHINE LEARNING					
VGG-19	DECISION TREE	GNB	KNN	RF	SVC	Metrics
93.14	92.22	63.86	72.99	95.77	74.99	A (%)
92.04	92.08	78.4	72.85	96.42	78.02	P (%)
94.22	92.64	39.79	74.64	95.21	70.64	R (%)
93.11	92.36	52.78	73.73	95.81	74.15	F1 score (%)
97.76	92.21	64.24	72.97	95.78	75.05	AUC (%)

Abbreviations: CNNs = convolutional neural networks; VGG-19 = visual geometry group; GNB = GaussianNB; KNNs = K-Nearest Neighbors; RF = Random Forest; SVC = Scikit-Learn; A = accuracy; P = precision; R = recall; AUC = area under the curve.

## Data Availability

The dataset used in this study was obtained from a Malaria Project of the National Library of Medicine (https://lhncbc.nlm.nih.gov/LHC-research/LHC-projects/image-processing/malaria-datasheet.html accessed on 7 May 2024). The data obtained can be obtained upon request from the corresponding author.

## Appendix A

**Table A1:** Results K-5-Fold cross-validation.

MACHINE LEARNING					
DECISION TREE Mean (SD)	GNB Mean (SD)	KNN Mean(SD)N	RF Mean (SD)	SVC Mean (SD)	Metrics
92.82 (0.53)	65.55 (0.92)	71.63 (0.34)	95.52 (0.33)	73.88 (0.76)	A (%)
93.12 (0.17)	77.89 (1.64)	70.99 (0.3)	96.28 (0.32)	75.66 (0.79)	P (%)
92.85 (1.01)	44.0 (3.75)	73.58 (0.79)	94.8 (0.68)	70.72 (0.85)	R (%)
92.95 (0.39)	56.09 (2.84)	72.26 (0.42)	95.56 (0.35)	73.11 (0.8)	F1 score (%)
92.89 (0.38)	73.74 (0.41)	78.14 (0.45)	98.66 (0.17)	86.67 (0.48)	AUC (%)

Abbreviations: GNB = GaussianNB; KNNs = K-Nearest Neighbors; RF = Random Forest; SVC = Support Vector Machine; SD = standard deviation; A = accuracy; P = precision; R = recall; AUC = area under the curve.
